# TLR–CD40 Cross-Talk in Anti-Leishmanial Immune Response

**DOI:** 10.3389/fimmu.2014.00220

**Published:** 2014-05-16

**Authors:** Himanshu Singh Chandel, Surya Prakash Pandey, Sayoni Roy, Noelle Doyen, Bhaskar Saha

**Affiliations:** ^1^National Centre for Cell Science, Pune, India; ^2^Pasteur Institute, Paris, France

**Keywords:** Toll-like receptors, PAMPs, CD40, *Leishmania*, immunomodulation, macrophages, dendritic cells, co-stimulatory molecules

Toll-like receptors (TLRs) recognize pathogen-associated molecular patterns (PAMPs) and activate innate immune cells to induce cytokines and co-stimulatory molecules such as CD40 and to enhance antigen presentation to T cells (1) that, upon activation, can either eliminate or support the pathogen (2). Herein, we propose that this duality in TLR functions results from their cross-talk with CD40. While all TLRs enhance CD40 expression, CD40 augments the expression of only TLR9 (3). As both CD40 and TLR9 induce expression of IL-12, a cytokine that induces the IFN-γ secreting Th1 cell differentiation (4), the CD40–TLR9 cross-regulation implies a positive feedback loop. By contrast, TLR1–TLR2 heterodimer down-regulates TLR9 expression (5) and antagonizes the development of Th1 response but favors the differentiation of regulatory T (T-reg) cells (Pandey et al., unpublished observation). Low CD40 expression levels in dendritic cells also promote T-reg cell differentiation (6). This duality can emerge from the sharing of signaling molecules. CD40 induces TRAF6-mediated, ERK-1/2-dependent IL-10 (7), which can inhibit the TLR-induced p38-MAPK activation and IL-12 production, antagonizing Th1 development. CD40-induced TRAF3-dependent p38-MAPK activation (7) can synergize with the TLR-activated p38-MAPK-dependent IL-12 production and Th1 differentiation. Using *Leishmania* infection, we show that the TLR–CD40 cross-talk can induce contrasting anti-leishmanial immune responses.

*Leishmania*, a protozoan parasite, lives in macrophages. *Leishmania* expresses lipophosphoglycan (LPG), proteoglycans, flagellin, and profilin for possible recognition by the host cell-expressed TLRs. Recognition of the *Leishmania*-expressed PAMPs results in differential immune responses, which can either reduce or exacerbate *Leishmania* infection. As TLRs modulate the expression of CD40, a co-stimulatory molecule whose expression levels modulate anti-leishmanial T cell responses, we propose that TLR–CD40 cross-talk significantly regulate the outcome of an anti-leishmanial immune response.

## Toll-Like Receptors Present Significant Diversity to Immunoregulation

A pathogen is perceived as a “danger” when specific molecular patterns associated with it [PAMPs or damage-associated molecular patterns (DAMPs)] are recognized by a set of TLRs, the mammalian homologs of toll, the anti-fungal resistance-mediating receptor in *Drosophila* (8, 9). Of the 13 TLRs, TLR10 is not expressed in mice whereas TLR11, TLR12, and TLR13 are absent from human (10). The extracellular domain of TLRs contains leucine-rich repeats (LRRs) arranged in an alpha-helix and a beta-pleated sheet. The LRR-rich loops impart the flexibility to this domain required for accommodating wide variety of chemically different PAMPs (11). The intracellular C-terminal domain has a toll/interleukin-1 receptor motif responsible for TLR signaling (12). Some TLRs – TLR1, TLR2, TLR4, TLR5, TLR6, TLR10, TLR11, TLR12 g – are located on cell surface to recognize the PAMPs on pathogen surface. Other TLRs – TLR3, TLR7, TLR8, TLR9, TLR13 – are located intracellularly on endosomes, lysosomes, and endoplasmic reticulum (13) to recognize the nucleic acids from the degraded pathogen (14). Thus, PAMPs on pathogen surface are first recognized by the TLRs on host cell surface. Once the pathogen is internalized and degraded, the released nucleic acids are recognized by the intracellular TLRs.

**Figure 1:**
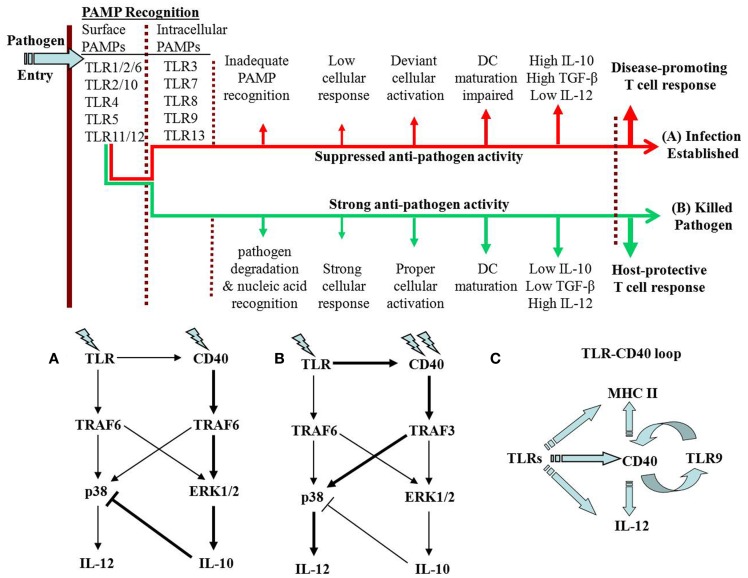
**TLR–CD40 cross-talk may result in one of two alternative possibilities**. As the pathogen enters its host, it is recognized by cell surface TLRs. Depending on the PAMP–TLR interaction, the TLR may trigger signals with one of two possible fates of the pathogen. **(A)** In case of anti-inflammatory responses, the pathogen degradation inside the cell is impaired leading to less release of the pathogen nucleic acids. As a result, the intracellular TLRs are not optimally activated. The immune response against the pathogen is suppressed causing establishment of the infection. In case of *Leishmania* infection, CD40 signaling through p38-MAPK is suppressed. **(B)** Alternatively, where the signaling results in pro-inflammatory response, the intracellular pathogen is degraded and the intracellular TLRs are optimally activated. In *L. major* infection, the host-protective function of CD40 involves strong p38-MAPK activation with resultant IL-12-dependent Th1 response. The thicker arrows in **(A,B)** represent the dominant signaling. **(C)** Possible feedback loops are proposed. TLRs increase CD40 expression but CD40 enhances the expression of only TLR9, an intracellular TLR that recognizes CpG motifs in pathogen DNA. This can be viewed as a positive feedback loop for enhancing IL-12 production and Th1 response. The other arm of the loop is the TLR-activated MHC-II expression, which is linked to CD40 expression and DC maturation. This is also required for a stronger and prolonged immune response against a pathogen.

Recognition of the PAMPs by the TLRs on host cell surface triggers intracellular signaling that may result in one of the two contrasting outcomes (Figure 1, top panel). The *Leishmania major* parasites that express low levels of LPG are unable to reduce TLR9 expression and are eliminated by the macrophages (5). By contrast, the virulent parasites express higher levels of LPG, reduce TLR9 expression, and survive in macrophages (5). The lipoprotein analogs with modified acylations are preferentially recognized by TLR1 (15). Thus, pathogens may modify PAMPs that differentially bind to the TLRs on host cell surface and signal to modulate the expression and function of intracellular TLRs. Differential signaling may result in either elimination or growth of the intracellular pathogen. Isolation of different strains expressing modified PAMPs and assessments of immune response to those modified PAMPs are required to verify this hypothesis.

## CD40 Plays Dual Immunoregulatory Roles in *Leishmania* Infection

CD40 is expressed on macrophages, dendritic cells, inflamed tissue histiocytes, and endothelial cells (16). CD40 signals though NF-κB to regulate the production of IL-12 (17), a pro-inflammatory cytokine required for Th1 differentiation (4). IL-4 is the cytokine that is required for the differentiation of Th2 cells, which produce IL-4, IL-5, and IL-13 (18). As a function of the strength of its stimulation, CD40 induces ERK-1/2-dependent IL-10 production (7). IL-10 expressed under MHC class-II promoter but not under IL-2 promoter aggravated *Leishmania* infection suggesting that the macrophage or the dendritic cell expressed IL-10 inhibited Th1 response (19). In *L. major* infection, the fate of the parasite is determined not only by Th1/Th2 balance but also by T-reg cells (6, 20–22) that produce IL-10, inhibiting Th1 differentiation but promoting infection (20–22). While low levels of CD40 expression on dendritic cells are required for T-reg cell expression (6), blockade of CD40–CD40L interaction on myeloid-derived suppressor cells (MDSCs) suppresses expansion of T-reg cells (23) suggesting CD40-induced dual regulation of T-reg cells.

It is shown that in response to higher doses of its ligand, CD40 signals from the cholesterol-rich domain through lyn, PKC-β, and p38-MAPK to induce IL-12 production whereas in response to lower doses of the ligand, the same receptor signals from the cholesterol-poor domain through syk, PKC-ζ, and ERK-1/2 to induce IL-10 production; ERK-1/2 inhibition results in enhanced activation of p38-MAPK and vice versa (7, 24–26). CD40 signals reciprocally through a bimodularly arranged cascade of kinases, wherein the reciprocity is incorporated by two feedback loops between p38-MAPK and syk and between ERK-1/2 and lyn (26). Thus, although the mechanism of the duality in CD40 functions is established, how CD40 regulates the contrasting fates of T-reg cells remains to be elucidated.

## Functional Plasticity in TLRs

Toll-like receptors bind to their respective ligands and dimerize before recruiting the adaptor molecules – MyD88, TIRAP/MAL, TRIF, and TRAM. MyD88 and TIRAP/MAL belong to the MyD88-dependent pathway and signal through NF-κB. TRIF and TRAM constitute the MyD88-independent pathway. Only TLR3 signals through MyD88-independent pathway and only TLR4 signals through both pathways (27). TRAF6 is another adaptor that mediates the TLR signals (27). The signals finally converge on MAPKs and activate different transcription factors that effectuate the gene expressions (27). TLRs are differentially involved in T cell activation and T-reg cell development. For example, the T cell-expressed TLR4 promotes the suppressive function of T-reg cells whereas TLR6 abrogates its suppressive function (28). Thus, TLR4 and TLR6 act antagonistically to each other in regulating T-reg cell functions. By contrast, TLR2 alone plays contradictory roles in T-reg cell expansion and in its suppressive function (29–31).

Among the TLRs, TLR2 represents a unique receptor, as it heterodimerizes with TLR1 or TLR6 or with TLR10, in human (32). The heterodimers broaden the repertoire of PAMPs recognized and may elicit different effector functions, which can even be counteractive. Some TLR2 ligands – arabinosylated lipoarabinomannan and lipoteichoic acid – induce pro-inflammatory responses (33, 34) but LPG, another TLR2 ligand, induces anti-inflammatory responses (5, 35, 36). The difference may result from the nature of the heterodimers recognizing the PAMPs. The TLR1/TLR2 heterodimer induces pro-inflammatory response whereas the TLR2/TLR6 heterodimer induces anti-inflammatory response or *vice versa* [(37); Pandey et al., unpublished results]. Besides forming heterodimers, TLR2 may form homodimer too. For example, SitC, a triacylated lipoprotein from *Staphylococcus aureus*, can induce cytokine response in the TLR1/TLR6-deficient macrophages (38). Although TLR2–TLR10 hetero-dimerization is a theoretical possibility, it appears unlikely because neither mice nor macrophages express TLR10. Although TLR1 and TLR6 cannot possibly recognize ligands or trigger signals on their own, their relative levels of expressions in a cell can determine the constitution of the predominant TLR2 heterodimer. The increased TLR2 expression in *L. major*-infected macrophages promotes TLR2 homo-dimerization, which is accentuated due to reduced TLR2–TLR6 association (Pandey et al., unpublished observation). In this case, because TLR2 homodimers are predominant and recruit primarily MyD88, TLR1–TLR2 and TLR2–TLR6 heterodimers may not be able to recruit enough MyD88. As the MAPKs and the transcription factors mediate TLR signaling (3, 39–41), the specificity, amplitude, and nature of the response will thus depend on the relative usage of these signaling intermediates. Thus, the plasticity in the TLR2-mediated recognition of PAMPs and elicitation of immune responses depend on the variations in the chemical structures of PAMPs, nature of TLR–PAMP interaction, recruitment of adaptor molecules, and competition between the TLRs for the available adaptor molecules.

## TLR and CD40 Cross-Talk Determines the Nature of Immune Responses

The response to an infection starts with the recognition of the PAMPs, perhaps, by multiple TLRs in tandem. Given the wide variety of PAMPs they recognize, the most probable TLRs to operate in tandem are TLR1, TLR2, TLR6, TLR10, and TLR4. In case of flagellated pathogens, TLR5 may recognize flagellin. The advantage of simultaneous trigger from TLR2 and TLR3 or TLR4 is that both MyD88-dependent and MyD88-independent pathways are involved increasing the overall strength and repertoire of TLR-derived signals. The combinations of TLRs may thus decide the nature of the signal and final effector functions (42) such as CD40 expressions that link the innate immune response to the adaptive immune response.

In peritoneal macrophages, CD40 expression in response to poly-I:C, LPS, and CpG, the TLR3, TLR4, and TLR9 ligands, respectively, is substantially enhanced, whereas CD40 stimulation enhances the expression of only TLR9 (3). *L. major* DNA induces IL-12 through TLR9 (43). CpG and CD40-ligand induced more IL-12 production from macrophages (3) and splenic dendritic cells (44) than that induced by either agent alone. On the other hand, low strength CD40 signal may synergize with the signal from TLR1–TLR2 heterodimers to strongly induce IL-10, which can inhibit p38-MAPK activation (Figure 1A). The CD40-induced IL-10 self-limits the CD40-induced p38-MAPK activation and anti-leishmanial functions (24). A possible feedback that comes into play in this CD40–TLR synergy is the quenching of TRAF6 availability to CD40 to result in less CD40-induced IL-10 production and relieving the autocrine IL-10 mediated inhibition of CD40-induced p38-MAPK activation and IL-12 production. Alternatively, exhaustion of TRAF6 by simultaneous signaling by multiple TLRs may divert a strong CD40 signaling primarily through TRAF3 to result in p38-MAPK activation and IL-12 production (Figure 1B). Thus, the enhanced IL-12 production as a result of TLR9 and CD40 synergy may represent a positive feedback loop between TLR9 and CD40 (Figure 1C). These reports imply that the TLR–CD40 cross-talk modulates the ensuing adaptive immune response.

Several reports support that TLRs can modulate CD40-mediated activation of adaptive immune system. PAMPs induce DC maturation by up-regulating MHC-II, CD40, and CD80/CD86 expressions (45) that are required for robust T cell responses. Because the binding of intracellular MHC-II with Btk via CD40 is required for sustained TLR activation, MHC-II deficiency impaired the TLR-induced production of pro-inflammatory cytokines and type-I interferon in macrophages and DC (46). CpG supported the survival and maturation of human plasmacytoid DC and, in synergy with CD40, induced T cells polarization to Th1 cells (47). Combined stimulation through TLR7 and CD40-induced CD8^+^ T cells expansion more than that observed with either agent alone (48). These reports indicate that CD40 and TLRs synergize to affect DC maturation, activation, survival, antigen presentation, and differentiation of CD4^+^ and CD8^+^ T cells.

## TLR–CD40 Cross-Talk as a New Paradigm for Immunoregulation

The TLR–CD40 cross-talk exemplifies that one of the fundamental physiological principles of maintaining homeostasis is the plasticity in receiving and processing signals. The signals from TLRs and CD40 modulate each other’s expression. Both receptors possess signaling plasticity modulating a range of effector functions (Figure 1) that affect both innate and adaptive immune systems. As pathogens sequentially involve cell surface and intracellular TLRs, the collective TLR activation or inhibition determines the CD40 expression levels. These evidences prompt a new model for the evolution of immune response. According to this model, TLR activation influences CD40 expression and signaling, resulting in both TLR and CD40 simultaneously signaling in the later phase of PAMP-induced innate immune response. As CD40 enhances TLR9 expression, TLR9, perhaps, through induction of IL-12 or further increase in CD40 expression, may further modulate the T cell response. Thus, a continued feedback between the TLR and CD40 during an immune response may finally decide the outcome of an infection. However, further verification of this model awaits detailed investigation.

## Conflict of Interest Statement

The authors declare that the research was conducted in the absence of any commercial or financial relationships that could be construed as a potential conflict of interest.

## References

[1] Iwasaki A Medzhitov R Toll-like receptor control of the adaptive immune responses. Nat Immunol (2004) 5:987–95 10.1038/ni1112 15454922

[2] Masopust D Picker LJ Hidden memories: frontline memory T cells and early pathogen interception. J Immunol (2012) 188:5811–7 10.4049/jimmunol.1102695 22675215 PMC3375618

[3] Chandel HS Pandey SP Shukla D Lalsare K Selvaraj SK Jha MK TLRs and CD40 modulate each others expression affecting *Leishmania major* infection. Clin Exp Immunol (2014) 176:283–90 10.1111/cei.12264 24387292 PMC3992041

[4] Manetti R Parronchi P Giudizi MG Piccinni MP Maggi E Trinchieri G Natural killer cell stimulatory factor induces T helper type 1 (Th1)-specific immune responses and inhibits the development of IL-4-producing Th cells. J Exp Med (1993) 177:1199–204 10.1084/jem.177.4.1199 8096238 PMC2190961

[5] Srivastava S Pandey SP Jha MK Chandel HS Saha B *Leishmania* expressed lipophosphoglycan interacts with toll-like receptor (TLR)-2 to decrease TLR-9 expression and reduce anti-leishmanial responses. Clin Exp Immunol (2013) 172:403–9 10.1111/cei.12074 23600828 PMC3646439

[6] Martin S Agarwal R Murugaiyan G Saha B CD40 expression levels modulate regulatory T cells in *Leishmania donovani* infection. J Immunol (2010) 185:551–9 10.4049/jimmunol.0902206 20525887

[7] Rub A Dey R Jadhav M Kamat R Chakkaramakkil S Majumdar S Cholesterol depletion associated with *Leishmania major* infection alters macrophage CD40 signalosome composition and effector function. Nat Immunol (2009) 10:273–80 10.1038/ni.1705 19198591

[8] Mills KH TLR-dependent T cell activation in autoimmunity. Nat Rev Immunol (2011) 11:807–22 10.1038/nri3095 22094985

[9] Lemaitre B Nicolas E Michaut L Reichhart JM Hoffmann JA The dorsoventral regulatory gene cassette spatzle/toll/cactus controls the potent antifungal response in *Drosophila* adults. Cell (1996) 86:973–83 10.1016/S0092-8674(00)80172-5 8808632

[10] Kawai T Akira S Toll-like receptors and their crosstalk with other innate receptors in infection and immunity. Immunity (2011) 34:637–50 10.1016/j.immuni.2011.05.006 21616434

[11] Jin MS Lee JO Structures of the toll-like receptor family and its ligand complexes. Immunity (2008) 29:182–91 10.1016/j.immuni.2008.07.007 18701082

[12] Bowie A O’Neill LA The interleukin-1 receptor/toll-like receptor superfamily: signal generators for pro-inflammatory interleukins and microbial products. J Leukoc Biol (2000) 67:508–14 10770283 10.1002/jlb.67.4.508

[13] Blasius AL Beutler B Intracellular toll-like receptors. Immunity (2010) 32:305–15 10.1016/j.immuni.2010.03.012 20346772

[14] Kawai T Akira S The role of pattern-recognition receptors in innate immunity: update on toll-like receptors. Nat Immunol (2010) 11:373–84 10.1038/ni.1863 20404851

[15] Takeuchi O Sato S Horiuchi T Hoshino K Takeda K Dong Z Role of toll-like receptor 1 in mediating immune response to microbial lipoproteins. J Immunol (2002) 169:10–4 10.4049/jimmunol.169.1.10 12077222

[16] van Kooten C Banchereau J Functions of CD40 on B cells, dendritic cells and other cells. Curr Opin Immunol (1997) 9:330–7 10.1016/S0952-7915(97)80078-7 9203418

[17] Yanagawa Y Onoé K Distinct regulation of CD40-mediated interleukin-6 and interleukin-12 productions via mitogen-activated protein kinase and nuclear factor kappaB-inducing kinase in mature dendritic cells. Immunology (2006) 117:526–35 10.1111/j.1365-2567.2006.02329.x 16556267 PMC1782254

[18] Bix M Locksley RM Independent and epigenetic regulation of the interleukin-4 alleles in CD4+ T cells. Science (1998) 281:1352–4 10.1126/science.281.5381.1352 9721100

[19] Groux H Cottrez F Rouleau M Mauze S Antonenko S Hurst S A transgenic model to analyze the immunoregulatory role of IL-10 secreted by antigen-presenting cells. J Immunol (1999) 162:1723–9 9973435

[20] Suffia I Reckling SK Salay G Belkaid Y A role for CD103 in the retention of CD4+CD25+ Treg and control of *Leishmania major* infection. J Immunol (2005) 174:5444–55 10.4049/jimmunol.174.9.5444 15845457

[21] Campanelli AP Roselino AM Cavassani KA Pereira MS Mortara RA Brodskyn CI CD4+CD25+ T cells in skin lesions of patients with cutaneous leishmaniasis exhibit phenotypic and functional characteristics of natural regulatory T cells. J Infect Dis (2006) 193:1313–22 10.1086/502980 16586370

[22] Fiorentino DF Bond MW Mosmann TR Two types of mouse T helper cell. IV. Th2 clones secrete a factor that inhibits cytokine production by Th1 clones. J Exp Med (1989) 170:2081–95 10.1084/jem.170.6.2081 2531194 PMC2189521

[23] Pan PY Ma G Weber KJ Ozao-Choy J Wang G Yin B Immune stimulatory receptor CD40 is required for T-cell suppression and T regulatory cell activation mediated by myeloid-derived suppressor cells in cancer. Cancer Res (2010) 70:99–108 10.1158/0008-5472.CAN-09-1882 19996287 PMC2805053

[24] Mathur RK Awasthi A Wadhone P Ramanamurthy B Saha B Reciprocal CD40 signals through p38MAPK and ERK-1/2 induce counteracting immune responses. Nat Med (2004) 10:540–4 10.1038/nm1045 15107845

[25] Sudan R Srivastava N Pandey SP Majumdar S Saha B Reciprocal regulation of protein kinase C isoforms results in differential cellular responsiveness. J Immunol (2012) 188:2328–37 10.4049/jimmunol.1101678 22271653

[26] Sarma U Sareen A Maiti M Kamat V Sudan R Pahari S Modeling and experimental analyses reveals signaling plasticity in a bi-modular assembly of CD40 receptor activated kinases. PLoS One (2012) 7:e39898 10.1371/journal.pone.0039898 22815717 PMC3399835

[27] Akira S Uematsu S Takeuchi O Pathogen recognition and innate immunity. Cell (2006) 124:783–801 10.1016/j.cell.2006.02.015 16497588

[28] Jin B Sun T Yu XH Yang YX Yeo AE The effects of TLR activation on T-cell development and differentiation. Clin Dev Immunol (2012) 2012:836485 10.1155/2012/836485 22737174 PMC3376488

[29] Liu H Komai-Koma M Xu D Liew FY Toll-like receptor 2 signaling modulates the functions of CD4+ CD25+ regulatory T cells. Proc Natl Acad Sci U S A (2006) 103:7048–53 10.1073/pnas.0601554103 16632602 PMC1444884

[30] Sutmuller RP den Brok MH Kramer M Bennink EJ Toonen LW Kullberg BJ Toll-like receptor 2 controls expansion and function of regulatory T cells. J Clin Invest (2006) 116:485–94 10.1172/JCI25439 16424940 PMC1332026

[31] Filippi CM Ehrhardt K Estes EA Larsson P Oldham JE von Herrath MG TLR2 signaling improves immunoregulation to prevent type 1 diabetes. Eur J Immunol (2011) 41:1399–409 10.1002/eji.200939841 21469083 PMC3100206

[32] Guan Y Ranoa DR Jiang S Mutha SK Li X Baudry J Human TLRs 10 and 1 share common mechanisms of innate immune sensing but not signaling. J Immunol (2010) 184:5094–103 10.4049/jimmunol.0901888 20348427

[33] Bhattacharya P Bhattacharjee S Gupta G Majumder S Adhikari A Mukherjee A Arabinosylated lipoarabinomannan-mediated protection in visceral leishmaniasis through up-regulation of toll-like receptor 2 signaling: an immunoprophylactic approach. J Infect Dis (2010) 202:145–55 10.1086/653210 20500089

[34] Gambhir V Yildiz C Mulder R Siddiqui S Guzzo C Szewczuk M The TLR2 agonists lipoteichoic acid and Pam3CSK4 induce greater pro-inflammatory responses than inactivated *Mycobacterium butyricum*. Cell Immunol (2012) 280:101–7 10.1016/j.cellimm.2012.12.001 23298864

[35] Sing A Rost D Tvardovskaia N Roggenkamp A Wiedemann A Kirschning CJ Yersinia V-antigen exploits toll-like receptor 2 and CD14 for interleukin 10-mediated immunosuppression. J Exp Med (2002) 196:1017–24 10.1084/jem.20020908 12391013 PMC2194041

[36] Netea MG Sutmuller R Hermann C Van der Graaf CA Van der Meer JW van Krieken JH Toll-like receptor 2 suppresses immunity against *Candida albicans* through induction of IL-10 and regulatory T cells. J Immunol (2004) 172:3712–8 10.4049/jimmunol.172.6.3712 15004175

[37] Li J Lee DS Madrenas J Evolving bacterial envelopes and plasticity of TLR2-dependent responses: basic research and translational opportunities. Front Immunol (2013) 4:347 10.3389/fimmu.2013.00347 24191155 PMC3808894

[38] Muller P Muller-Anstett M Wagener J Gao Q Kaesler S Schaller M The *Staphylococcus aureus* lipoprotein SitC colocalizes with toll-like receptor 2 (TLR2) in murine keratinocytes and elicits intracellular TLR2 accumulation. Infect Immun (2010) 78:4243–50 10.1128/IAI.00538-10 20679445 PMC2950364

[39] Sumbayev VV Yasinska IM Role of MAP kinase-dependent apoptotic pathway in innate immune responses and viral infection. Scand J Immunol (2006) 63:391–400 10.1111/j.1365-3083.2006.001764.x 16764692

[40] Carneiro AB Iaciura BM Nohara LL Lopes CD Veas EM Mariano VS Lysophosphatidylcholine triggers TLR2- and TLR4-mediated signaling pathways but counteracts LPS-induced NO synthesis in peritoneal macrophages by inhibiting NF-κB translocation and MAPK/ERK phosphorylation. PLoS One (2013) 8:e76233 10.1371/journal.pone.0076233 24312681 PMC3848743

[41] McGuire VA Gray A Monk CE Santos SG Lee K Aubareda A Cross talk between the Akt and p38α pathways in macrophages downstream of toll-like receptor signaling. Mol Cell Biol (2013) 33:4152–65 10.1128/MCB.01691-12 23979601 PMC3811899

[42] Sanchez PJ McWilliams JA Haluszczak C Yagita H Kedl RM Combined TLR/CD40 stimulation mediates potent cellular immunity by regulating dendritic cell expression of CD70 in vivo. J Immunol (2007) 178:1564–72 10.4049/jimmunol.178.3.1564 17237405

[43] Abou Fakher FH Rachinel N Klimczak M Louis J Doyen N TLR9-dependent activation of dendritic cells by DNA from *Leishmania major* favors Th1 cell development and the resolution of lesions. J Immunol (2009) 182:1386–96 10.4049/jimmunol.182.3.1386 19155485

[44] Krug A Towarowski A Britsch S Rothenfusser S Hornung V Bals R Toll-like receptor expression reveals CpG DNA as a unique microbial stimulus for plasmacytoid dendritic cells which synergizes with CD40 ligand to induce high amounts of IL-12. Eur J Immunol (2001) 31:3026–37 10.1002/1521-4141(2001010)31:10<3026::AID-IMMU3026>3.0.CO;2-H 11592079

[45] Koch F Stanzl U Jennewein P Janke K Heufler C Kampgen E High level IL-12 production by murine dendritic cells: upregulation via MHC class II and CD40 molecules and downregulation by IL-4 and IL-10. J Exp Med (1996) 184:741–6 10.1084/jem.184.2.741 8760828 PMC2192732

[46] Liu X Zhan Z Li D Xu L Ma F Zhang P Intracellular MHC class II molecules promote TLR-triggered innate immune responses by maintaining activation of the kinase Btk. Nat Immunol (2011) 12:416–24 10.1038/ni.2015 21441935

[47] Cella M Scheidegger D Palmer-Lehmann K Lane P Lanzavecchia A Alber G Ligation of CD40 on dendritic cells triggers production of high levels of interleukin-12 and enhances T cell stimulatory capacity: T-T help via APC activation. J ExpbreakMed (1996) 184:747–52 10.1084/jem.184.2.747 8760829 PMC2192696

[48] Ahonen CL Doxsee CL McGurran SM Riter TR Wade WF Barth RJ Combined TLR and CD40 triggering induces potent CD8+ T cell expansion with variable dependence on type I IFN. J Exp Med (2004) 199:775–84 10.1084/jem.20031591 15007094 PMC2212721

